# Certain Forms of Menorrhagia and Treatment of the Same

**Published:** 1889-04

**Authors:** W. D. Bizzell

**Affiliations:** Atlanta, Ga.; Professor Principles and Practice of Medicine, Southern Medical College


THE ATLANTA MEDICAL AND SURGICAL JOURNAL.

VOL. VI. APRIL, 1889. No. 2.

### Original Communications.

### CERTAIN FORMS OF MENORRHAGIA AND TREATMENT OF THE SAME.*

BY W. D. BIZZELL, M.D., ATLANTA.

Professor Principles and Practice of Medicine, Southern Medical College.

Menorrhagia is generally understood to mean either an abnormal increase in quantity of blood lost within the normal menstrual period, or an undue prolongation as to time, the total amount of blood lost in either case being much beyond what it should be for the individual. In the majority of cases both of these elements are present, the flow is relatively too free and unduly prolonged.

The knowledge that menstruation is a recurring and necessary phenomenon in the female during the period of sexual activity, is a fact as old as the race.

While it is true we have much more exact knowledge than was possessed by the profession previous to the studies of Bis-

*Read at the meeting of the Southern Surgical and Gynecological Association, Birmingham, Ala., Dec. 1888.

choff, Pfleüger and Robt. Lee, and others of more recent date, yet many of the questions connected with the function are not so completely or clearly answered as is desirable. The doctrine enunciated and maintained by these writers, and accepted by the profession, that periodical ovulation, or the development and discharge of ova from the Graffian follicle, accompanied and determined the menstrual flux, must in a light of fuller knowledge be revised.

First, ovulation does not always accompany the menstral flux, but may precede or follow it.

Second, following the removal of both ovaries, whether from disease or by Battey's operation, the menstrual flow does not in all cases immediately cease, but recurs at more or less regular intervals for a considerable period, sometimes for several years.

More recently Lawson Tait has maintained that the fallopian tubes and not the ovaries determined menstruation, and the flow is induced by active movements of the tubes to grasp the ovary in the fimbræ, and for this reason has insisted, that removal of the appendages is necessary to accomplish what Battey proposed when he evolved his original operation. Even this more radical operation does not in all cases immediately arrest the periodical flow. Dr. Mary Putnam Jacobi, in a series of elaborately written articles, (Amer. Jour. Obste., 1885), maintains as the result of careful study in a large number of living subjects, and many sections of the cadaver, at varying periods, menstrual and intermenstrual, that the womb is in a state of pre-menstrual progressive preparation for the flux or pregnancy, either of which may be considered the climax of preparatory effort. Or the flow having ceased, a period of post-menstrual retrogression follows until the womb reaches the minimum weight and vascularity of the quiescent mid-menstrual period, which state of quiescence constitutes only about one third of the time between periods.

The pre-menstrual preparatory development, according to Dr. Jacobi, consists in "enlargement by growth of the periuterine and ovarian sinuses." "The essential and efficient cause of the menstrual hemorrhage lies in the accumulation of blood in the periuterine and ovarian sinuses."

This accumulation is not a congestion. Up to this point the whole process is one of physiological preparation for the reception and development of a fertilized germ or ovum. She says: "When fecundation has not occurred, the growing endometrium arrived at a certain point of development, is then arrested by the non-expansion of the uterine cavity. The opposing surfaces of the endometrium touch, press against each other; it exfoliates, laying bare the surface capillary loops, which break at some point, so that capillary oozing begins."

The foregoing studies and deductions harmonize thoroughly with the conclusions of Englemann, Kundrot and more recently Johnstone,* that the endometrium is in no sense to be regarded as a mucous membrane, being devoid of basement membrane. On the contrary it is an adenoid or glandular structure, the glandular follicles and the intervening space being sparsely covered with rounded cells, which have only a remote resemblance to epithelium, and are attached directly to the capillaries and musculature of the endometrium, between which the glandular follicles penetrate.

The dominating and controlling influence which the nervous system maintains over all the functions of the body, must not be lost sight of in the consideration of menstruation, either from the physiological or pathological standpoint.

If at the proper age none of the signs of puberty appear, and none of the peculiar nervous and circulatory disturbances described, as the menstrual molimen assert themselves, the ovaries will be found absent or undeveloped. If the accessory organs, breasts, pudendum, etc., are developed, and the menstrual molimen is absent or feebly expressed, the vagina will be either absent or short and narrow, and the womb either absent, or rudimentary. If the molimen is well pronounced, together with the evidences of well-developed womanhood, and there is no flow, there is either atresia vaginæ or absence or slight development of the uterus.

*Trans. British Gynecology Association, Vol. vii, No. 7.

The first step in the processes which inaugurate the menstrual flow is, development of the ovaries and the ripening and discharge of mature ova. Accompanying, or accessory, to this is the periodical enlargement of the uterine and ovarian sinuses, vaso-motor impulse, filling the sinuses, and, lastly, the visible flow, most likely as described by Dr. Putnam Jacobi.

It does not necessarily follow, that the menses would immediately cease to appear after the removal of the ovaries, even on theoretical grounds. The vaso-motor and trophic cyclic impulse once thoroughly imparted to the organism, would dominate the uterus, in the usual fashion, and the flow would appear at intervals of more or less regularity, for a considerable period; the impulse growing weaker, the pre-menstrual trophic charges would not be sufficient to provoke the flow and it would cease to appear.

These trophic changes, and the hyperæmia, and attendant flow, amounting in all to only a few ounces, when the womb, ovaries, appendages and accessory organs are healthy, may, under certain circumstances, be very much in excess of the normal quantity and unduly prolonged. The only wonder is that enlarged sinuses, permanent hyperæmia and excessive flow does not oftener follow or replace the normal physiological recurring hyperæmia.

Exposure to cold, excessive exertion, or fatigue, worry and excitement during a menstrual period may develop the necessary excessive engorgement, a repetition of these events in a careless or reckless subject, the morbid condition becomes fixed on the victim. This type of cases are frequently encountered in strong and plethoric young women, who think they can endure all things, and survive any imprudence.

Occasionally, in delicate subjects, with feeble blood-making powers, the same imprudence will develop menorrhagia, but in this type we are far more apt to have, after one or two excessive periods, dysmenorrhœa with scanty flow.

In young married women, during the child-bearing period, by far the most frequent cause is imperfect evolution of the womb, following an abortion or labor at term. Whatever the cause producing the sub-involution, whether rapid and successive child-bearing, too early rising after parturition, rupture of the cervix, imperfect removal of secundines, forceps delivery with rupture of perineum or dragging down and dislocation of the pelvic facia, suppression of the lochia, sepsis; anything which causes determination of blood toward the pelvic organs, will give rise to that condition we all recognize as sub-involution. A heavy womb enlarged in all dimensions, a patiulous gaping os, often with mucous membrane everted and exhibiting fungosities, the sound penetrating to a depth of 3½ to 5½ inches, from a half to one and a half inches deeper than we would expect in metritis, where the uterus appeared to be of same size as determined bi-manually.

The condition of the womb is one of softening and relaxation of the muscular fibres, which are infiltrated with fat, sinuses enlarged, blood-vessels of the endometrium very much enlarged, requiring only the additional vascularity of the menstrual molimen and incident development to produce a flow far exceeding the natural amount, and often times amounting to alarming hemorrhage.

The treatment of these various conditions producing menorrhagia, is either general or local.

In young girls, where there is a full habit, and the menorrhagia has followed exposure to cold, excessive fatigue, dancing, long walks, horse-back exercise, etc. during a period, simple hygienic treatment, of plain food, absence of excitement, fresh air, loose stays, well protected body, thick shoes, regular bowels, occasional salines and the supine position during the menstrual period, will, if faithfully carried out, in a surprisingly short time effect a cure in the vast majority of cases. When this fails and local treatment is required, the application, once a week to the endometrium of Battey's solution of iodine of carbolic acid, together with tampons of boroglyceride and glycerine, equal parts, renewed every second day, should be tried.

Shop-women, particularly those who have borne children, in consequence of constantly standing upon their feet, even during the menstrual period, suffer from engorgement of the womb, menorrhagia and more or less leucorrhœa. In this type careful tamponage of the vagina, with glycerine and iodoform cotton, using just a sufficient amount to slightly elevate the uterus, together with at least partial rest during the menstrual periods, will usually effect a cure, unless the nervous system is undermined and general nutrition has suffered considerable impairment. If so a season of complete rest, tonics and general farridization may be required to perfect the cure.

In cases of sub-involution following miscarriage or child-birth at term, a careful examination should be made to determine the local condition, as a proper selection of procedure will determine whether the cure shall be speedy and safe. If a laceration of the cervix is discovered and is extensive, it should be closed. In some cases a careful curetting should precede the operation by from five to seven days, especially should this be done in case the laceration is of long standing and the menorrhagia has followed a more recent miscarriage. In recent cases, where the laceration has resulted from an instrumental delivery, followed by arrest of lactation and tardy convalescence, the simple operation of trachelorrhophy is followed by involution and cure.

The vast majority of cases presenting sub-involution and memorrhagia, will be found on examination not to have suffered laceration of the cervix, but we find that condition typical of sub-involution. Relaxed muscles gorged with fat, enlarged sinuses; blood-vessels of the endometrium enlarged with thin walls, bleeding under the slightest provocation, the mere passage of a sound being sufficient to start more or less free oozing. If the organ is fairly movable without much tenderness of surrounding tissues, the case is much simplified.

A certain proportion of these cases may in time spontaneously subside, and a certain number may be aided to recovery by internal medication, iron, arsenic, ergot, etc., together with proper hygiène. But in the majority of cases without local treatment a state of general anæmia with hyperæsthetic hysteria will ensue, and the restoration to health will be much retarded.

The choice of method in the local treatment is a matter of much importance. The philosophy underlying all treatment is to give increased tone to the muscular structure, remove fungusities or fragments of secundries, and by obliteration, or contraction of caliber, diminish the passive hyperæmia of the endometrium.

The curette, first given to us by Recamier, and endorsed by Sims, has, after a season of comparative neglect, in recent years been the chosen method of treatment. If this method is adopted it will be found that the dull curette of Thomas, is only suitable in a minority of cases; it is true, the sharp curette in careless hands may do damage, though, as Dr. Gill Wylie* says: "A man who cannot use the sharp curette without inflicting injury, had best not undertake the treatment at all."

The application of the fuming nitric acid to the endometrium, so strongly commended and successfully practiced by the late Lombe Athill, is when properly applied, a safe and speedy method of cure in many cases. If only partially or inefficiently used, it will cause further determination of blood to the uterus, and the tendency to hæmorrhage will be increased. When properly applied the acid destroys by erosion and thrombosis, quickens the circulation in the deeper structure of the uterus, stimulates the muscles to contract, hastens absorption of fat and other effete material, and the womb is soon restored to its proper size and proportions.

The thorough use of the curette, and the proper use of the acid, produce in their mechanical and other effects on the endometrium an almost identical effect. With better, more available and safer methods, I think the use of the acid should be abandoned.

By the curette, adherent residual masses of placenta are removed, fungosities and small senile growths of the endometrium scraped away, and varicosities destroyed, but unless there results a stimulating tonic effect upon the muscular structure and deeper circulation of the uterus, the fungosities will surely be reproduced, the hemorrhages will again recur, and the operation must be repeated again and again.

For the past two years I have treated all cases of this character coming under my observation after the method devised and practiced by the late Dr. Taliaferro†, of Atlanta, viz., by the application of the intra-uterine tampon.

*American System of Gynecology, vol. 1.

†Transactions Georgia Medical Association, 1886.

Of course, Sims' and many others had used small tampons of cotton lint, etc., charged with various medicinal agents, passed up and left within the uterical cavity. But Dr. Taliaferro was the first to advocate the application of the tampon, firmly packed within the uterus, to promote disgorgement of vessels, quicken absorption, restore healthy tone to the uterine structure, much as we bring about repair by bandaging varicose ulcer of the leg.

As Dr. Taliaferro,* in another communication, truly says: "As a therapeutic measure, the intra-uterine tampon is simple, safe and marvelous. With the proper observation of the contra-indications to all intra-uterine applications, the remedy is perfectly safe. It of course should not be used when there is the vestige of inflammation in the peri-uterine structure.

"So long as there is sufficient oozing of blood to saturate the tampon, it is removed and re-applied daily; otherwise it remains for two days."

"If iodoform is used with the packing, as should always be done, the patient is secure against sepsis, the dressings removed being clean and free from odor, though it remain for two or three days."

Dr. Taliaferro's method is to place the patient in the knee-chest or Sims position; as a rule the knee-chest is to be preferred. The perineum, then firmly lifted or retracted by Sims' speculum, is held by an assistant; a strong tenaculum, or, what is better, a spring-catch vulsellum forceps, is used to seize the anterior lip of the womb, is drawn down toward the vulva and firmly held by the assistant or the left hand of the operator.

The tampon, which should be made of a roll of sample or absorbent cotton, drawn out and rolled between the hands until moderately firm, is wrapped spirally with a sewing thread and tied at the lower end; about four to six inches of the thread being left to facilitate extraction. Into this tampon iodoform is thorougly rubbed, or, what is better, it is dipped in a saturated solution of iodoform in ether, and the ether allowed to evaporate. I generally keep, enclosed in a cannister, a supply of the

*ATLANTA MEDICAL AND SURGICAL JOURNAL, Oct. 1886.

rolls, of various sizes and lengths, already prepared by dipping and drying.

The patient being in proper position as already described, the tampon is seized at its distal extremity by a slender pair of dressing forceps, (I generally prefer Thomas' intra-uterine,) and carried through the cervix and to the fundus uteri; the blades are then slightly separated and withdrawn a couple of inches, when the tampon is again seized and another section crowded in; this is repeated until the entire uterine cavity is snugly packed, which may in some cases require two or three rolls. Powdered iodoform is then dusted over the cervix and vagina and a tampon of glycerine and iodoform is passed up to the cervix, back of this is placed a moderate tamponage of cotton, unless there be at the time a very free flow of blood from the womb, which it is desirable to stop at once, when a firm vaginal tamponage will be required to supplement and reinforce the intra-uterine tampon.

Under the firm pressure of the intra-uterine tamponage, those tissue changes of construction and destruction and absorption go forward, whereby the womb reaches a normal condition. When the muscular tone of the womb has been restored the tampon will no longer be tolerated, but will be forced out. A light glycerine and iodoform vaginal tampon should be worn for a week or ten days after the discontinuance of the intra-uterine packing. It is best to confine the patient to bed at least for the first few days during the use of the tampon, and to the house and very moderate exercise the whole period of treatment, otherwise development of hæmatocile or localized peritonitis may force a discontinuance of the treatment.

At the October meeting of the *Societe Medicale de Geneva*, Professor Vuillet presented a paper on "Progressive and Continuous Dilatation of the Cervix and Uterine Cavity," by cotton tampons, for ocular inspection and diagnostic purposes, using small pledgets of iodoform cotton from the size of a pea to an almond. He carries these tampons up to the cervix with slender dressing forceps, and with a stiff metallic rod forces the cotton into the cervical canal, continuing the dilatation if necessary with bougies.

"He plugs the uterine cavity as a dentist plugs a carious tooth. These are renewed at the end of twenty-four or forty-eight hours." Vuillet only makes use of this method when it is necessary or any benefit to the patient. "When the introduction of the finger or curette suffices for the treatment, dilatation is pushed only to the extent necessary for that purpose," etc. Showing that this distinguished teacher did not adopt his treatment as a curative measure or perceive its value for this purpose.

It is mainly to again call the attention of the profession to this wonderfully effective treatment, as practiced by Dr. Taliaferro, that I have written these lines. I am satisfied that those who are making opinions and authority for the present generation and history for the future, do not fully appreciate this resource, or it would find an abiding place among the resources of gynecology.

Since this paper was written I have read with some interest an article by Dr. Eugene C. Gerhung*, of St. Louis, Mo., presented to the American Gynecological Society, Sept., 1888: "Repression of Menstruation as a Curative Agent in Gynecology." I have been very much interested in his reasoning and conclusions, which may be summarized thus: That in a case of memorrhagia, where there can be no doubt that the great blood losses are surely undermining the patient's health, the physician is not only justified in endeavoring to diminish the amount of blood lost at the next menstrual period, but even by his art *prevent the flow altogether*. I confess I was a little disappointed when I read his method and the means by which he proposed to accomplish these beneficent results, which was tamponing the vagina with absorbent cotton, wrung out of a one to two per cent. aqueous solution of alum, packed bit by bit so as to make a tight vaginal tampon, under this plan the loss of blood is insignificant. As to the intra-uterine tampon, he says in a given case: "The intra-uterine tampon made matters worse." If Dr. Gerhung will begin his treatment by pushing his first tampon, after the manner of Dr. Taliaferro, firmly against the fundus, and continuing until the womb is entirely filled with elastic aseptic cotton, and then

*American Journal Obstetrics, Nov., 1888.

place the vaginal tampon, he can control the oozing, however free it may be.

As to the correctness and propriety of his proposition to restrain the menstrual flow in the sense of almost arresting it, temporarily, there is no question of doubt. When I begin the use of the intra-uterine tampon, I do not consider the approach of menstruation as any bar to its continued use, but go forward with the method until I have accomplished that for which I have inaugurated it.

